# A multimodal meta-analysis of gray matter alterations in trigeminal neuralgia

**DOI:** 10.3389/fneur.2023.1179896

**Published:** 2023-08-03

**Authors:** Xiang Huang, Boyi Li, Yuming Li, Junyu Lin, Huifang Shang, Jing Yang

**Affiliations:** ^1^Department of Neurology, West China Hospital of Sichuan University, Chengdu, Sichuan, China; ^2^Department of Radiology, West China Hospital of Sichuan University, Chengdu, Sichuan, China

**Keywords:** trigeminal neuralgia, functional MRI, voxel-based morphometry, multimodal, meta-analysis

## Abstract

**Background:**

Brain gray matter alterations in patients with trigeminal neuralgia (TN) have been detected in prior neuroimaging studies, but the results are heterogeneous. The current study conducted coordinate-based meta-analyses across neuroimaging studies, aiming to find the pattern of brain anatomic and functional alterations in patients with TN.

**Methods:**

We performed a systematic literature search of PubMed, Embase, and Web of Science to identify relevant publications. A multimodal meta-analysis for whole-brain voxel-based morphometry (VBM) studies and functional imaging studies in TN was performed using anisotropic effect size-based signed differential mapping.

**Results:**

The meta-analysis comprised 10 VBM studies with 398 TN patients and 275 healthy controls, and 13 functional magnetic resonance imaging studies with 307 TN patients and 264 healthy controls. The multimodal meta-analysis showed conjoint structural and functional brain alterations in the right fusiform gyrus and inferior temporal gyrus, bilateral thalamus, left superior temporal gyrus, left insula, and inferior frontal gyrus. The unimodal meta-analysis showed decreased gray matter volume alone in the left putamen, left postcentral gyrus, and right amygdala as well as only functional abnormalities in the left cerebellum, bilateral precuneus, and left middle temporal gyrus.

**Conclusion:**

This meta-analysis revealed overlapping anatomic and functional gray matter abnormalities in patients with TN, which may help provide new insights into the neuropathology and potential treatment biomarkers of TN.

## Introduction

Trigeminal neuralgia (TN) is a common cranial neuralgia, which is featured by recurrent severe paroxysmal, electric shock-like, lancinating pain limited to one or more of the trigeminal nerve branches, lasting from a fraction of a second to 2 min per episode and is triggered by innocuous stimuli (1). TN can be classified into secondary TN which is caused by a neurological disease other than neurovascular compression, and primary TN which includes classical TN (cTN) which develops without apparent cause other than neurovascular compression and idiopathic TN (iTN) without significant abnormalities in either electrophysiological tests or conventional magnetic resonance imaging (MRI) (1). The annual incidence of TN is estimated to be four to five per 100,000 and affects one in 15,000–20,000 people worldwide with a female preponderance (2). Increased depression, anxiety, and poor sleep were also reported in patients with TN, indicating that TN is a debilitating neuropathic pain condition that affects human physiological and psychological activities (3). Although the origin of TN is considered to be at the trigeminal root (4), the peripheral mechanisms alone could not entirely explain the pathophysiology of TN, especially iTN.

By changing the flow and integration of information through many brain regions, chronic pain including TN could influence brain function and behavior (5). Central facilitation and central allodynic mechanisms that engage the nociceptive neurons at the trigeminal nucleus, thalamic, and cortical levels were suggested to be related to this persistent pain (6). Growing evidence with advanced neuroimaging techniques has demonstrated that both the brain’s structural and functional changes may also contribute to the development of TN. Voxel-based morphometry (VBM) is a hypothesis-free technique that allows voxel-wise comparisons of the whole brain tissue volume between groups *in vivo* (7). VBM studies have reported decreased or increased gray matter volume (GMV) in various brain regions in patients with TN, including the secondary somatosensory cortex, insula, thalamus, anterior cingulate cortex, cerebellum, caudate nucleus, amygdala, postcentral gyrus, and precuneus compared to healthy controls (HCs) (8–15), while functional studies using function MRI also detected increased or decreased brain activation in the thalamus, postcentral gyrus, cerebellum, anterior cingulate cortex, and other cortical and subcortical brain regions (10, 11, 16, 17). However, these structural and functional studies often reported different or even contradictory results. The reason for these inconsistencies might be associated with differences in design, imaging methodology, and sample sizes, as well as heterogeneities in demographic and clinical characteristics across studies.

A better understanding of the brain alterations in subjects with TN may provide new insight into the pathophysiology of primary TN, which may contribute to facilitating advances in treatment. In this study, we conducted a multimodal meta-analysis to identify the conjoint and dissociated brain changes in the structure and function of subjects with primary TN. Although there was one meta-analysis that included anatomic and functional neuroimaging studies in subjects with TN published before August 2018, it did not conduct a multimodal meta-analysis to examine the overlapping regions between anatomic and functional brain alterations, and many related studies have been published since then (18). In this study, we integrated the recently published VBM and functional studies on primary TN and conducted unimodal and multimodal meta-analyses by using anisotropic effect size-based signed differential mapping (AES-SDM), which is a powerful coordinate-based meta-analytic tool widely applied in neurological diseases (19). In addition, meta-regression analyses were performed to evaluate potential relationships between brain GMV and functional changes and clinical features.

## Materials and methods

### Search strategy and study selection

The review process was based on the Preferred Reporting Items for Systematic Reviews and Meta-Analyses (PRISMA) statement. A systematic search was conducted in the Web of Science, PubMed, and Embase up to October 3rd, 2022, using the following keywords (“trigeminal neuralgia” OR “trifacial neuralgia”) AND (“magnetic resonance imaging” OR “MRI” OR “functional MRI” OR “fMRI”). We also searched the reference lists of included studies to obtain potential studies.

### Inclusion/exclusion criteria

**Figure 1:**
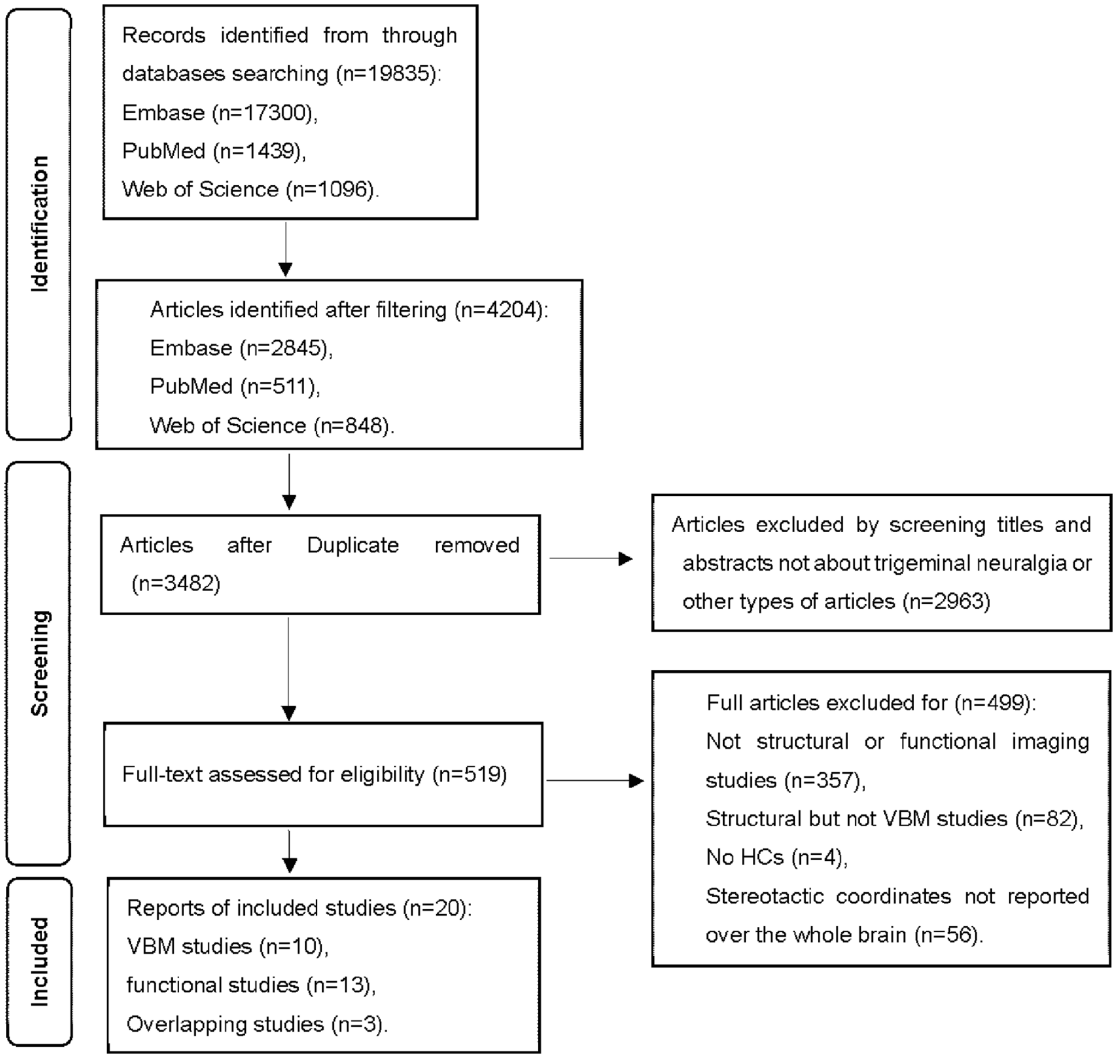
Flowchart of the identification of the meta-analysis.

The inclusion criteria for the candidate studies were: (a) published in English with peer review; (b) employed whole-brain functional imaging or structural imaging using VBM to explore gray matter changes in primary TN compared to HCs; (c) localized effects using Montreal Neurological Institute (MNI) or Talairach coordinates; (d) used thresholds for significance corrected for multiple comparisons or uncorrected with spatial extent thresholds. These studies were considered ineligible: (a) review articles, case reports, and meta-analyses; (b) studies that exclusively used a region of interest approach; (c) studies where the whole-brain results in three-dimensional coordinates could not be retrieved after contacting corresponding authors by emails. Group coordinates were regarded as separate datasets if a study contains more than one independent patient sample. Figure 1 summarizes the study selection procedures.

### Data extraction

Study selection and data extraction were conducted in a standard form by two investigators separately and any disagreements were assessed by the third author. Information, such as peak coordinates (x, y, z) and effect sizes (t-, z-, or *p*-values) of structural or functional differences between subjects with primary TN and HCs were extracted from each dataset according to the SDM tutorial.

### Quality assessment

To better evaluate the quality of included studies, a 10-point checklist (Supplementary Table S1) was applied according to prior meta-analysis (20). This assessment evaluated both the demographic and clinical features of each study’s sample and the imaging methodology applied in each study. Each item on the checklist received a score of 0, 0.5, or 1 according to the criteria that were not met, partially met, or fully met, respectively. The checklist paid attention to rating the completeness of the included studies, not to criticize the investigators or the work itself.

### Meta-analyses of structural and functional studies

Voxel-wise meta-analyses of regional brain differences in gray matter structure and functional activity were performed independently using the AES-SDM software package (version 5.15, www.sdmproject.com). The procedure has been described in detail in a previous study (21) and briefly summarized here. Using an anisotropic non-normalized Gaussian kernel, AES-SDM recreates an effect-size map and an effect-size variance map of the signed structural or functional activity differences between groups from peak coordinates and effect sizes for each study. All maps were then combined as a mean map with a standard-effects model weighted by the sample size, intra-study variability, and inter-study heterogeneity. Default SDM kernel size and statistical thresholds [full width at half maximum (FWHM) = 20 mm, voxel *p* = 0.005, peak height z = 1, and cluster extent = 10 voxels] were used (22).

### Multimodal analysis

Both structural and functional findings were summarized in a single meta-analytic map as described in a previous study (23, 24) to detect the brain regions showing functional and structural changes. A multimodal meta-analysis in AES-SDM was conducted to assess brain regions of overlapping functional and anatomic alterations between primary TN subjects and HCs by computing the value of *p* overlap within each voxel from the original meta-analytic maps accounting for error (details in Supplementary materials). A more stringent probability threshold was applied for this multimodal analysis (*p* < 0.0025) than that used in unimodal meta-analyses (23).

### Heterogeneity, publication bias, and sensitivity analysis

To detect the between-study heterogeneity of individual clusters in the meta-analytical results, Q-statistic with a random-effects model and tested for significance with a permutation approach (uncorrected *p* < 0.005) was applied; thresholds (voxel threshold *p* < 0.005, peak Z > 1, and cluster extent of 10 voxels) indicated significant heterogeneity (22). We also applied the I^2^ statistic tests to quantify the degree of heterogeneity. Funnel plots were created by Egger’s tests for visual inspection, in which any result with *p* < 0.05 indicated obvious publication bias. Jackknife sensitivity analyses were performed to assess the robustness of the main meta-analytical findings by iteratively repeating analyses excluding one dataset each time. Additionally, subgroup analyses of those studies applying same-strength MRI scanners, same smoothing kernel, and statistical parametric mapping (SPM) software were performed. These findings are considered well replicable if the results are still significant in all or majority of the combinations of datasets.

### Meta-regression analyses

To explore the potential effects of clinical variables (e.g., age and disease duration) on regional brain anatomic or functional alterations in subjects with primary TN, meta-regression analyses were conducted using a stringent threshold (*p* < 0.0005 and extent threshold = 10 voxels).

## Results

### Literature results

Twenty studies (8–14, 16, 17, 25–35) were finally included in the current meta-analyses. Among these studies, three studies (11, 13, 33) reported both structural and functional results. Three fMRI studies (16, 17, 29) shared the same sample but reported results with different imaging measures, while another two pairs of fMRI studies (13, 26, 27, 31) examined another same dataset with different imaging measures. One fMRI study and one VBM study divided patients into two subgroups and conducted two comparisons respectively, so two sets of data were obtained from each of these two studies (11, 12). Another VBM study divided patients into three groups according to different symptoms and conducted three comparisons (34). In the end, we obtained 14 datasets from the 10 VBM studies comprising 398 subjects with primary TN and 275 HCs and 10 datasets from the 13 fMRI studies comprising subjects with 307 primary TN and 264 HCs. The details of demographic and clinical features and quality assessments of the included studies are presented in Table 1. Supplementary Table S2 shows the detailed quality assessment scores of the included studies, all of which got relatively high scores.

**Table 1:** Characteristics of included structural and functional studies in the meta-analysis.

Study	Type	No. of subjects (F)	Age (years)	Disease duration (years)	Software Package/imaging modality	Scanner (T)	FWHM (mm)	Threshold	Quality scores
VBM studies									
(25)	TN	21(17)	55(2.1)	8.5(2.1)	SPM5/T1	3.0	6	*p* < 0.01, corrected	8.5
	HC	30(24)	53.6(3.2)						
(8)	TN	60(36)	62(13.2)	8.3(6.7)	SPM8/T1	1.5	8	*p* < 0.05, corrected	9
	HC	31(11)	61.8(9)						
(9)	TN	28(13)	45.86(11.17)	8.43(3.65)	SPM8/T1	1.5	8	*p* < 0.05, corrected	9
	HC	14(9)	44.89(7.67)						
(11)	TN(R)	36 (20)	58.0 (7.7)	5.80 (6.28)	SPM8/T1	3.0	8	*p* < 0.05, corrected	10
	TN(L)	26 (18)	59.0(6.6)	5.27 (4.92)					
	HC	19 (15)	55.6 (8.2)						
(13)	TN	29 (19)	48.14 (11.89)	6.02 (4.35)	SPM12/T1	3.0	6	*p* < 0.05, corrected	10
	HC	34 (21)	43.32 (10.07)						
(14)	TN	40 (23)	55.76 (8.23)	7.08 (5.29)	SPM12/T1	3.0	8	*p* < 0.05, corrected	9
	HC	40 (23)	55.80 (8.09)						
(12)	pTNwNVC	23 (11)	53.30 (8.66)	5.74 (3.35)	SPM12/T1	3.0	8	*p* < 0.05, corrected	9
	pTNwoNVC	22 (14)	47.77 (9.24)	4.97 (2.09)					
	HC	45 (23)	49.36 (11.58)						
(32)	TN	30 (21)	66.24 (10.57)	6.84 (6.81)	FSL/ T1	1.5	NA	*p* < 0.05, corrected	10
	HC	15 (10)	62.14 (10.61)						
(33)	TN	34 (18)	53.06 (10.91)	4.63 (3.53)	SPM12/T1	3.0	6	*p* < 0.05, corrected	10
	HC	29 (14)	54.21 (6.33)						
(34)	TN1	16 (10)	59.1 (5.2)	1.1 (0.7)	SPM12/T1	3.0	8	*p* < 0.005, corrected	9
	TN2	17 (11)	60.5 (10.0)	4.8 (1.5)					
	TN3	16 (9)	63.6 (6.8)	15.1 (5.5)					
	HC	18 (11)	59.8 (8.0)						
Functional studies									
(26)	TN	17 (10)	63.41 ± 7.25	6.98 ± 5.64	SPM8/fMRI	1.5	6	*p* < 0.05, corrected	10
	HC	19 (10)	62.53 ± 7.41						
(10)	TN	38 (22)	55.87 (8.38)	7.05 (5.32)	REST/fMRI	3.0	8	*p* < 0.05, corrected	8.5
	HC	38 (22)	55.89 (8.06)						
(27)	TN	17 (10)	62.53 (7.41)	6.98 (5.64)	SPM8/fMRI	1.5	6	*p* < 0.05, corrected	10
	HC	19 (11)	61.75 (6.02)						
(11)	TN(R)	36 (20)	58.0 (7.7)	5.80 (6.28)	FSL/fMRI	3.0	6	*p* < 0.05, corrected	10
	TN(L)	26 (18)	59.0 (6.6)	5.27 (4.92)					
	HC	19 (15)	55.6 (8.2)						
(28)	TN	23 (9)	59.6 (12.5)	5.69 (3.33)	SPM8/fMRI	3.0	4	*p* < 0.05, corrected	10
	HC	23 (11)	63.1 (9.8)						
(13)	TN	29 (19)	48.14 (11.89)	6.02 (4.35)	SPM12/fMRI	3.0	6	*p* < 0.05, corrected	10
	HC	34 (21)	43.32 (10.07)						
(17)	TN	28 (16)	51.392 (9.372)	3.733 (4.102)	SPM8/fMRI	3.0	6	*p* < 0.05, corrected	9.5
	HC	28 (16)	51.357 (9.302)						
(29)	TN	28 (16)	51.392 (9.372)	3.733 (4.102)	SPM8/fMRI	3.0	6	*p* < 0.01, corrected	9.5
	HC	28 (16)	51.357 (9.302)						
(30)	TN	28 (14)	37.4 (9.0)	4.5 (13.3)	SPM12/fMRI	3.0	8	*p* < 0.05, corrected	10
	HC	28 (14)	40.3 (10.3)						
(31)	TN	29 (19)	48.14 (11.89)	6.02 (4.35)	SPM12/fMRI	3.0	6	*p* < 0.05, corrected	10
	HC	34 (21)	43.32 (10.07)						
(16)	TN	28 (16)	51.392 (9.372)	3.733 (4.102)	SPM8/fMRI	3.0	NA	*p* < 0.05, corrected	10
	HC	28 (16)	51.357 (9.302)						
(33)	TN	34 (18)	53.06 (10.91)	4.63 (3.53)	REST/fMRI	3.0	6	*p* < 0.05, corrected	10
	HC	29 (14)	54.21 (6.33)						
(35)	TN	48 (28)	54.48 (10.35)	7.10 (5.43)	FSL/fMRI	3.0	8	*p* < 0.05, corrected	10
	HC	46 (27)	56.50 (8.23)						

FWHM, full-width-at-half-maximum; TN, trigeminal neuralgia; HC, healthy control; R, right; L, left; VBM, voxel-based morphometry; SPM, Statistical Parametric Mapping; T1, T1-weighted images; FSL, FMRIB’s Software Library; REST, RESTing state fMRI data analysis toolkit; fMRI, functional magnetic resonance imaging; pTNwNVC, primary trigeminal neuralgia with neurovascular compression; pTNwoNVC, primary trigeminal neuralgia without neurovascular compression; NA, not available.

### Regional alterations in gray matter volume and functional activity

**Figure 2:**
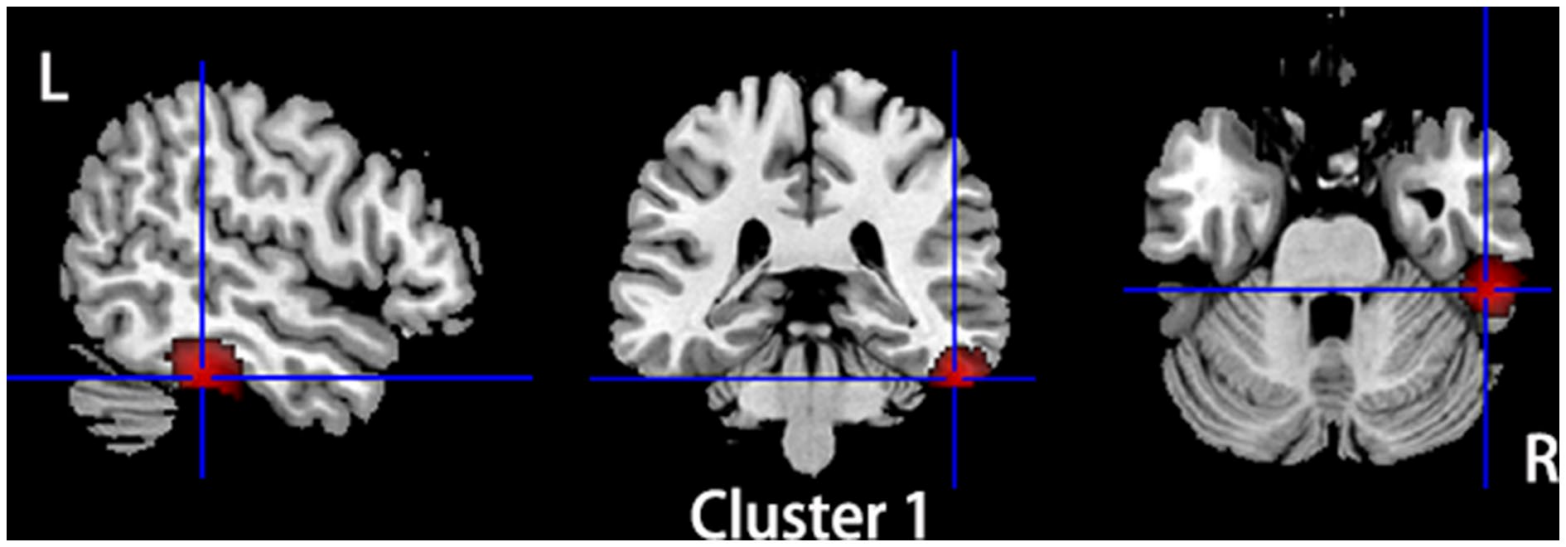
Regions of gray matter volume increase in patients with primary TN compared with healthy controls. Compared to healthy controls, patients with primary TN showed increased gray matter volume in the right inferior temporal gyrus and fusiform gyrus.

**Figure 3:**
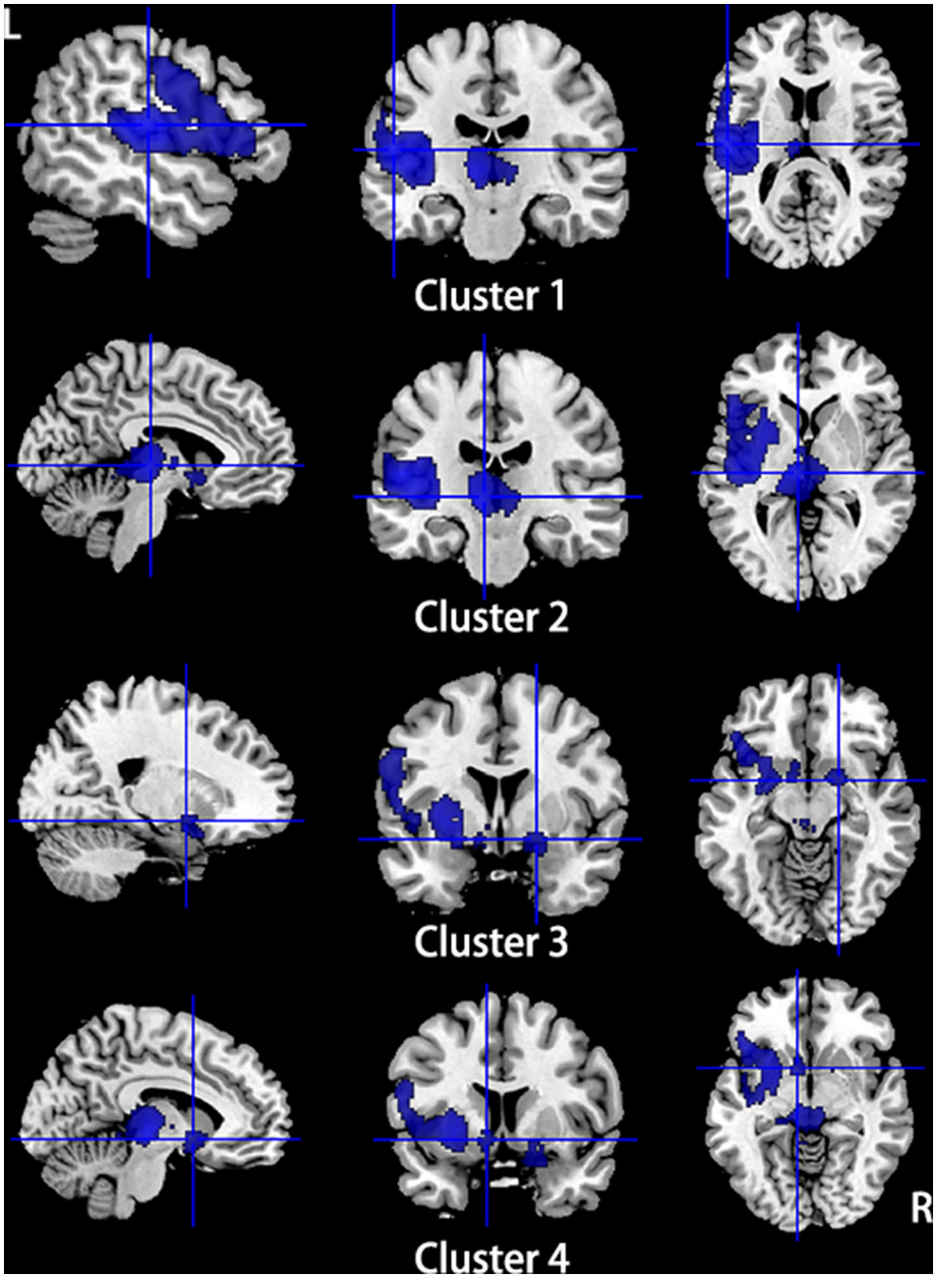
Regions of gray matter volume decrease in patients with primary TN compared with healthy controls. Compared to healthy controls, patients with primary TN showed decreased gray matter volume in the left insula, left superior temporal gyrus, left putamen, left postcentral gyrus, left inferior frontal gyrus (Cluster 1), bilateral thalamus (Cluster 2), right amygdala (Cluster 3), and left striatum (Cluster 4).

Compared to HCs, subjects with primary TN showed increased GMV in the right inferior temporal gyrus and fusiform gyrus, while they showed decreased GMV in the left insula, left superior temporal gyrus, left putamen, left postcentral gyrus, left inferior frontal gyrus, right amygdala, bilateral thalamus, and left striatum (Table 2; Figures 2, 3).

**Table 2:** Gray matter volume alterations in patients with primary trigeminal neuralgia compared with healthy controls.

Regions	No. of voxels	MNI coordinates (x, y, z)	SDM-Z score	*p*-value	Egger’s test(p)	I^2^	Clusters’ breakdown
TN > HC							
Cluster 1	476	52, −34, −28	1.189	<0.001	0.224	<0.001	Right inferior temporal gyrus
							Right fusiform gyrus
TN < HC							
Cluster 1	5,153	−52, −18, 14	−3.436	<0.001	0.989	<0.001	Left insula
							Left superior temporal gyrus
							Left putamen
							Left postcentral gyrus
							Left inferior frontal gyrus, opercular part
							Left inferior frontal gyrus, orbital part
Cluster 2	1,257	−6, −20, 2	−3.239	<0.001	0.528	<0.001	Left thalamus
							Right thalamus
Cluster 3	92	20, 2, −10	−2.156	0.001	0.448	<0.001	Right amygdala
Cluster 4	47	−8, 8, −6	−2.233	0.001	0.675	1.05%	Left striatum

TN, trigeminal neuralgia; MNI, Montreal Neurological Institute; SDM, Seed-based d Mapping; HCs, healthy controls; I^2^, percentage of variance attributable to study heterogeneity.

**Figure 4:**
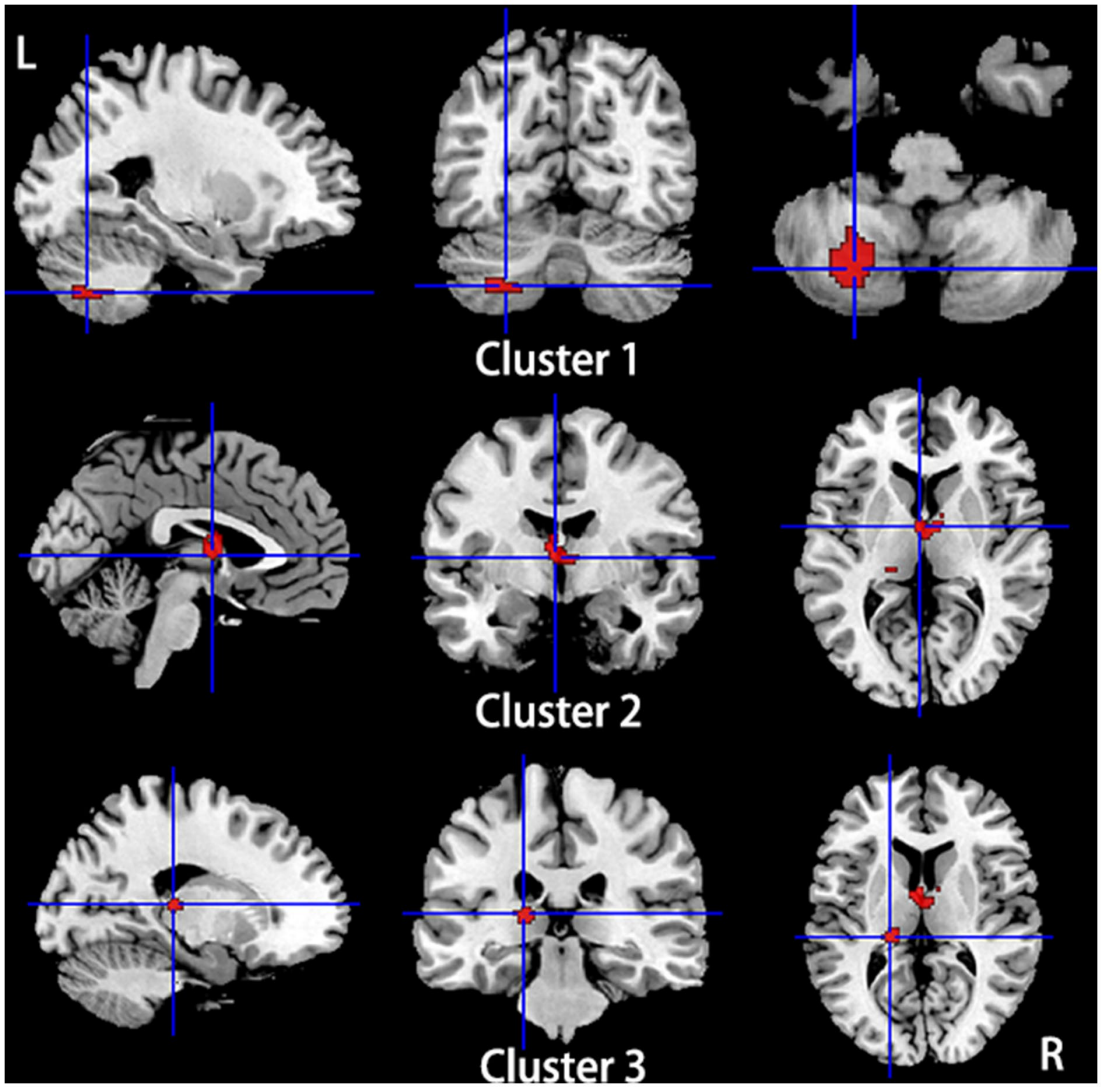
Regions of hyperactivity in patients with primary TN compared with healthy controls. Compared to healthy controls, patients with primary TN showed hyperactivity in the Left cerebellum, hemispheric lobule VIII (cluster 1), right thalamus (cluster 2), and left thalamus (cluster 3).

**Figure 5:**
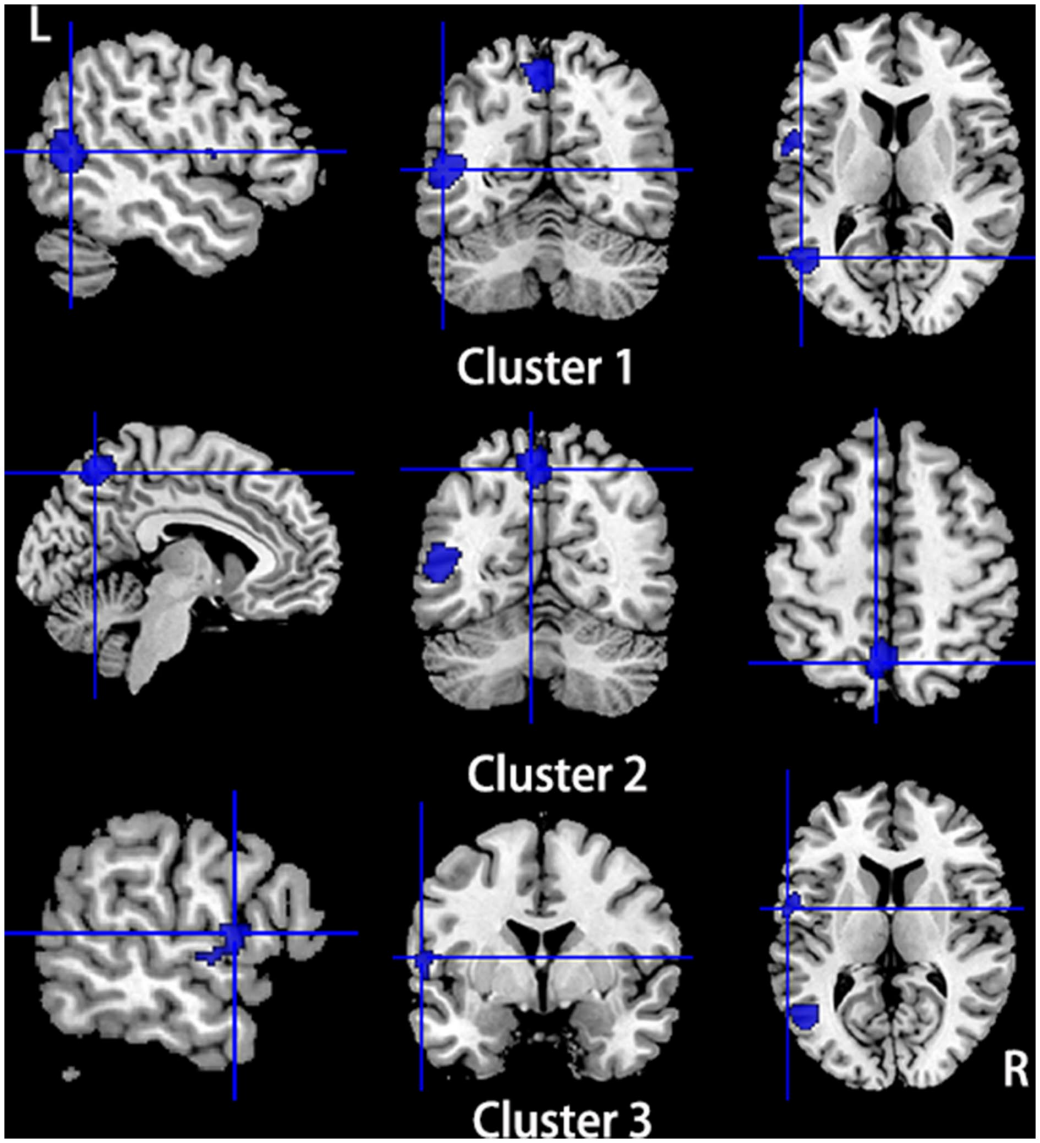
Regions of hypoactivity in patients with primary TN compared with healthy controls. Compared to healthy controls, patients with primary TN showed hypoactivity in the left middle temporal gyrus (cluster 1), bilateral precuneus (cluster 2), and left superior temporal gyrus (Cluster 3).

Subjects with primary TN showed hyperactivation in the left cerebellum, hemispheric lobule VIII, and bilateral thalamus, while they showed hypoactivation in the bilateral precuneus, left middle temporal gyrus, and superior temporal gyrus (Table 3; Figures 4, 5).

**Table 3:** Brain functional alterations in patients with primary trigeminal neuralgia compared with healthy controls.

Regions	No. of voxels	MNI coordinates (x, y, z)	SDM-Z score	*p*-value	Egger’s test(p)	I^2^	Clusters’ breakdown
TN > HCs							
Cluster1	123	−26, −66, −44	1.527	0.002	0.264	1.06%	Left cerebellum, hemispheric lobule VIII
Cluster 2	117	−4, −4, −6	1.637	0.001	0.438	3.63%	Right thalamus
Cluster 3	36	−18, −28,10	1.457	0.003	0.283	37.4%	Left thalamus
TN < HCs							
Cluster 1	293	−50, −60, 10	−1.500	0.002	0.638	26.6%	Left middle temporal gyrus
Cluster 2	273	−6, −62, 54	−1.781	<0.001	0.304	9.96%	Left precuneus
							Right precuneus
Cluster 3	127	0, −38, 42	−1.482	0.001	0.166	<0.001	Left superior temporal gyrus

TN, trigeminal neuralgia; MNI, Montreal Neurological Institute; SDM, Seed-based d Mapping; HCs, healthy controls; I^2^, percentage of variance attributable to study heterogeneity.

### Multimodal analysis of brain gray matter volume and functional activity

**Figure 6:**
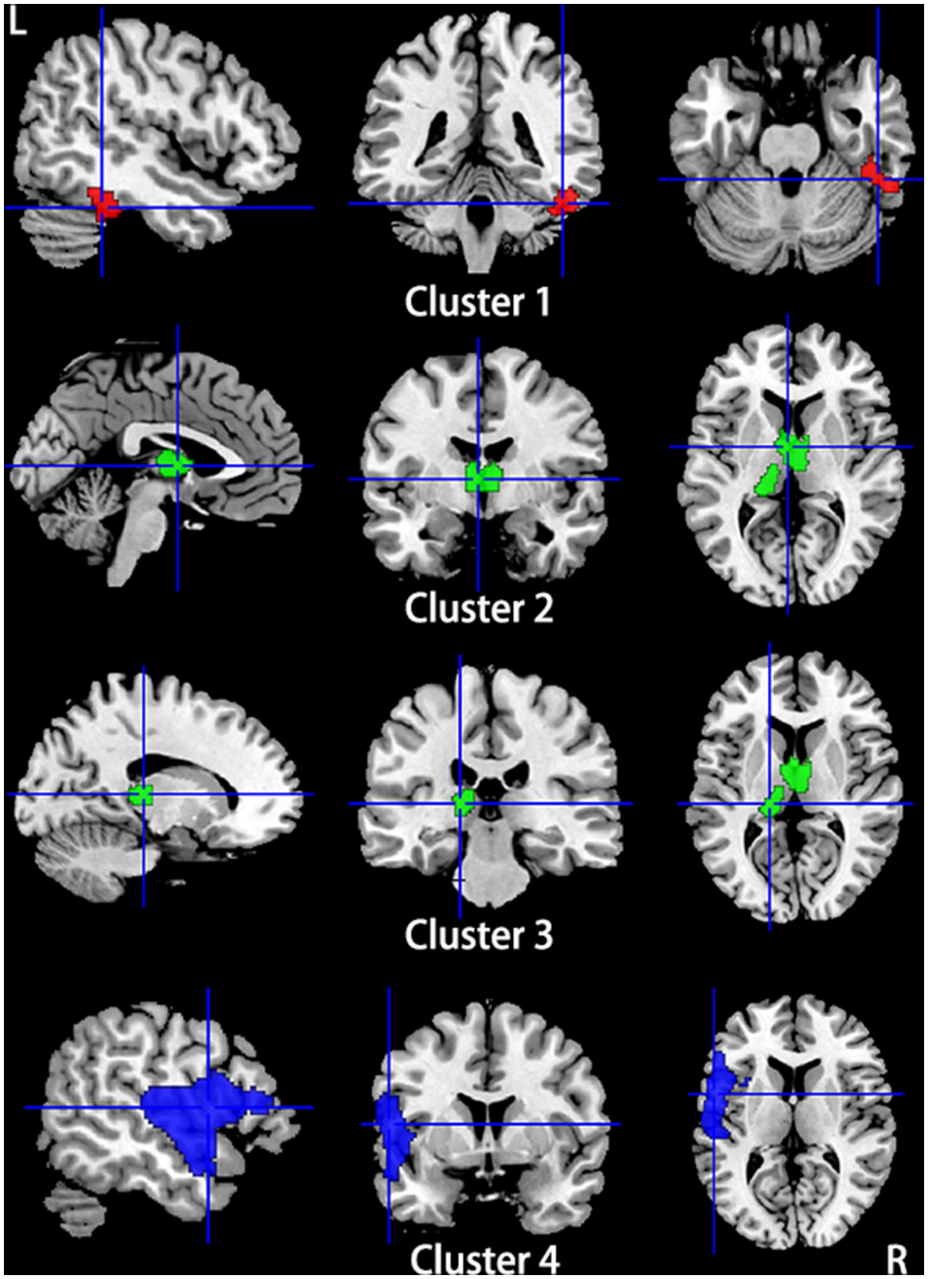
Regions showed conjoint gray matter structural and functional alterations in patients with primary TN compared with healthy controls from the multimodal meta-analysis. Compared to healthy controls, patients with primary TN showed increased gray matter volume and hyperactivity in the right inferior temporal gyrus and fusiform gyrus (Cluster 1), decreased gray matter volume and hyperactivity in the bilateral thalamus (Cluster 2) and left thalamus (cluster 3), and decreased gray matter volume and hypoactivity in left superior temporal gyrus and inferior frontal gyrus and left insula (Cluster 4).

Compared to HCs, subjects with primary TN showed a conjoint increase of GMV and functional activity in the right fusiform gyrus and right inferior temporal gyrus and a decrease of GMV and functional activity in the left superior temporal gyrus, left insula, and left inferior frontal gyrus. The bilateral thalamus showed gray matter hypotrophy with hyperactivity in subjects with primary TN compared to HCs (Table 4; Figure 6).

**Table 4:** Multimodal structural and functional abnormalities in patients with primary trigeminal neuralgia compared with healthy controls.

Cluster	MNI	Voxels	Cluster breakdown
Increased GMV + hyperactivity			
	48, −40, −26	284	Right fusiform gyrus
			Right inferior temporal gyrus
Increased GMV + hypoactivity			
(None)			
Decreased GMV + hyperactivity	−4, −4, 6	508	Bilateral thalamus
	−16, −28,8	248	Left thalamus
Decreased GMV + hypoactivity	−54, 4, 10	2,364	Left superior temporal gyrus
			Left insula
			Left inferior frontal gyrus, opercular part
			Left inferior frontal gyrus, triangular part

MNI, Montreal Neurological Institute; BA, Brodmann Area; GMV, gray matter volume.

### Sensitivity analysis, heterogeneity, and publication Bias analysis

The results from both VBM and functional meta-analyses were highly replicable according to the jackknife sensitivity analyses and subgroup analyses limited to methodologically homogenous groups of studies (Supplementary Tables S3, S4). Heterogeneity analysis using Q statistics indicated that there was no significant variability between studies for meta-analysis of VBM studies and meta-analysis of functional studies. The I^2^ statistics indicated substantial heterogeneity with an I^2^ of 37.4 and 26.6% for left thalamus and left middle temporal gyrus, respectively, in the functional meta-analysis, and low heterogeneity for all the other findings in both structural and functional meta-analysis (<10%; Tables 2, 3). No obvious asymmetry of all significant brain regions was unveiled from the funnel plots (Supplementary Figures S1–S11). Egger’s tests revealed no publication bias from the quantitative assessment measured (Tables 2, 3).

### Meta-regression results

The meta-regression analysis showed that age at scanning time was positively associated with decreased GMV in the left superior temporal gyrus and left striatum, while disease duration was positively associated with hypoactivation in the left thalamus.

## Discussion

To the best of our current knowledge, this is the largest multimodal neuroimaging meta-analytic study investigating overlapping and dissociated gray matter structural and functional changes in subjects with primary TN. We identified conjoint gray matter anatomic and functional alterations in the right fusiform gyrus and inferior temporal gyrus, bilateral thalamus, left superior temporal gyrus, left insula, and inferior frontal gyrus in subjects with primary TN compared to HCs. In addition, several brain regions including the left putamen, left postcentral gyrus, and right amygdala exhibited only gray matter atrophy, while the left cerebellum, bilateral precuneus, and left middle temporal gyrus showed only functional alterations. The current meta-analysis emphasized a central role in the pathophysiology of primary TN with both consistent anatomic and functional alterations mainly in brain regions associated with perception and the processing of pain and regulation of emotion.

An increase in GMV with hyperactive function was found in the right fusiform gyrus and inferior temporal gyrus in subjects with TN relative to HCs. The fusiform gyrus plays a role in sensory integration and cognitive processing (36). The right fusiform gyrus is also one of the visual processing centers of the mammalian brain, which works in integrating visual information and forming conscious perception (29). A previous study found that painful electrical shock may activate the fusiform cortex, indicating that pain in TN may also activate the fusiform gyrus (37). The structural and functional abnormalities in the right fusiform gyrus found in the current study emphasized the role of the fusiform cortex involved in pain perception and modulation in patients with TN. The inferior temporal gyrus has the projection to the amygdala and hippocampus, which plays a crucial role in emotional processing, regulation, and self-cognition (17, 38). Increased functional activity within this region may be relative to the inhibition of pain caused by negative emotions and cognitive degradation (17).

Gray matter atrophy with hyperactivation was detected in the bilateral thalamus in subjects with TN. The thalamus is a pivotal relay station for sensory information. Evidence from neuroimaging studies, physiological studies, and lesioning studies indicated that the thalamus may hold the key to pain consciousness and understanding of spontaneous and evoked pain in chronic pain conditions (39–41). Altered GMV and functional activity in the thalamus were also found in neuroimaging studies in other chronic pain conditions including migraine, chronic back pain, and fibromyalgia (41–43). Using multiple brain imaging techniques, significant volume loss in the somatosensory thalamus was found to be associated with hypoactivity in the thalamic reticular nucleus and primary somatosensory cortex in patients with TN. These findings suggested that altered thalamic structure and activity in chronic neuropathic pain may result in disturbed thalamocortical circuits (44). Neuropathic-specific hyperactivity in the thalamic neurons was found several days after injuring the trigeminal ganglion neurons in the animal model, indicating that these may contribute to neuroplastic changes in the thalamocortical circuits and produce long-lasting neuropathic pain in the orofacial region (45). The decreased GMV of the thalamus identified in the meta-analysis may be related to impaired processing and modulation of neuropathic pain signals, and the hyperactivation in the thalamus may be secondary to the gray matter atrophy and suggest increased sensory load in patients with TN.

A conjoint decrease in GMV and functional activity was found in the left inferior frontal gyrus, left insula, and superior temporal gyrus in subjects with TN. Anatomic and functional alterations in the left inferior frontal gyrus were also reported in other pain-related disorders such as myofascial-type temporomandibular disorders (46), diabetic neuropathic pain (47), and affective disorders (48). The left inferior frontal gyrus is a subregion of the prefrontal cortex, which is one of the key regions involved in the affective, cognitive, and emotional aspects of pain, and can be activated by pain stimuli and has clear implications in pain perception and modulation (49). GM atrophy with hypoactivity in the left inferior frontal gyrus may lead to the loss of inhibitory control of the nociceptive transmission system (50) and may provide the neuroanatomical basis for the increased incidence of mood disorders reported in TN (3). The insula is involved not only in sensorimotor and affective processing, autonomic information, and high-level cognition but also in pain perception and its modulation according to prior neuroimaging studies (51, 52). Changes in cortical gyrification and associated altered functional connectivity were identified in the left insula and its critical roles in the pathophysiology of TN were emphasized (53). The superior temporal gyrus is critical for extracting meaningful linguistic features from speech input and is involved in managing perception in auditory, speech, emotions, and comprehension (54). The temporal lobe including superior temporal gyrus plays a critical role in the perception of chronic pain (9, 10). The decreased GMV and functional activity in the left superior temporal gyrus may indicate an adaptative mechanism initiated in response to chronic pain and activity-dependent dendritic changes.

Additionally, we found gray matter atrophy in the right amygdala in the unimodal meta-analysis. The amygdala is a pivotal brain center for the emotional-affective dimension of pain and pain modulation. Other chronic pain conditions in previous studies also detected changes in anatomic and functional activities within the amygdala (55, 56). The amygdala as an essential part of the emotion-related network involved in emotional processing, anxiety, stress regulation, reward learning, and motivation is associated with affective disorders, including anxiety and depression (57). The structural and functional alterations within the amygdala may also contribute to a significantly increased incidence of anxiety and depression in patients with TN (3). Gray matter atrophy and altered connectivity in the amygdala may be exhibited during the transition to chronic pain. Using the multimodal neuroimaging approach to assess TN-related structural and functional brain alterations in emotion-related and pain-related networks (13), reduced GMV in the bilateral amygdala and three significantly altered amygdala-related functional circuits were found and they supported the pain-related and emotion-related network deficits in TN patients. In this study, we found asymmetric changes in structural changes within the amygdala, which may be related to the hemispheric lateralization of pain processing. The left central nucleus of the amygdala showed lower neuronal activity than the right in models of inflammatory pain, independent of the side of the peripheral injury (58). Meanwhile, asymmetrical time-dependent activation of bilateral amygdala neurons was reported in rats with peripheral neuropathy, with enhanced evoked activity in the right central nucleus of the amygdala persisting compared to short-term increases in activity in the left, indicating that the right central nucleus of the amygdala was highly involved in the processing of sensitization related to neuropathy (59). The asymmetric findings in the amygdala may be the case that the hypotrophy in the right amygdala may be resulting from persistent activity.

Several brain regions showed anatomic or functional anomalies alone in subjects with TN in the current study. The anatomic abnormalities included GMV atrophy in the left putamen and left postcentral gyrus, all without functional brain activity changes. The putamen is connected with both cortical regions involved in sensory processing, attention, and memory. Widespread decreases in pain-related brain activation were identified in patients with putamen lesions, suggesting that the putamen may be pivotally involved in the shaping of the pain experience (60). GMV reduction in the putamen in the current meta-analysis was consistent with previous studies (11, 15, 25, 34, 61), supporting the hypothesis that the putamen contributed to sensory aspects of pain. The postcentral gyrus is associated with the anticipation, intensity, discrimination, spatial, and temporal summation aspects of pain processing and self-reported pain intensity (15).

The hyperactivity in the left cerebellum and hypoactivity in the left middle temporal gyrus and bilateral precuneus were not accompanied by GMV alterations. The cerebellum plays a crucial role in modulating motor control and is involved in a range of movement disorders. Also, a previous study found that the cerebellum can receive extensive somatosensory input via spinocerebellar pathways, underscoring its additional role as a sensory organ (62). Cerebellar functional changes may result from increased sensory input derived from long-term and high-frequency inputs related to patients with TN. The left middle temporal gyrus is involved in the perception of pain as previous neuroimaging studies reported pain-related activation within this brain region (63, 64). Both the precuneus and left middle temporal gyrus are crucial brain regions of the default mode network (DMN). The DMN is associated with mediating the recognition and rumination of pain and internal processing including autobiographical memory, self-reference, and stimulus-independent thought (65). DMN connectivity was identified to be related to pain frequency and intensity in adolescents (66), and alterations in connectivity between the DMN and the pain network were reported in adults with chronic pain (67). Extending prior works, current findings indicated that TN may be related to the desegregation of the DMN, and the hypoactivity of critical nodes within this network may represent altered maintenance of attention and vigilance of pain and reflect TN as a background pain sensation (27).

There were also some limitations in this study. First, the heterogeneity among the included studies in demographic data and methodologies could not be entirely ruled out. Second, for structural alterations, studies that investigated the cortical thickness alterations using surface-based morphometry (SBM) in TN were not included because the meta-analysis of these studies needed to use a thickness mask (68) that is different from the one used for VBM studies, and the limited number of published SBM studies in TN prevents the conducting of the vertex-based meta-analysis (12, 69, 70). Third, various physiological bases and underpinning assumptions of the included methods used in the fMRI studies may affect the results of the meta-analysis, hindering the comprehensive overview of whole-brain functional alterations in TN. Lastly, the current multimodal meta-analysis aims to identify overlapping brain regions showing both anatomic and functional changes; the directionality and causality of the findings cannot be directly answered.

## Conclusion

Our multimodal meta-analysis demonstrated GM alterations in patients with TN, characterized by conjoint structural and functional alterations in the bilateral thalamus, left inferior frontal gyrus, insula and superior temporal gyrus, and right inferior temporal gyrus, and separative GMV reduction in the putamen, postcentral gyrus and amygdala, and altered function in cerebellum and nodes of the DMN. The pattern of conjoint and disassociated changes in the structure and function of GM prompted our understanding of the mechanisms underlying TN and may provide potential treatment biomarkers for TN in the future.

## Author contributions

XH: conception, organization, execution, data acquisition, statistical analysis, and manuscript preparation. BL: organization, execution, data acquisition, statistical analysis, and manuscript preparation and revision. YL: project execution, data acquisition, manuscript review, and critique. JL and HS: manuscript review and critique. JY: conception, organization, statistical analysis, manuscript review, and critique; he was responsible for the overall content as the guarantor. All authors contributed to the article and approved the submitted version.

## Funding

This study was supported by the National Natural Science Foundation of China (No. 81971071), the Applied Basic Research Programs of Science and Technology Department of Sichuan Province (No. 2021YJ0447), and the 1.3.5 project for disciplines of excellence-Clinical Research Incubation Project, West China Hospital, Sichuan University (No. 2021HXFH044).

## Conflict of interest

The authors declare that the research was conducted in the absence of any commercial or financial relationships that could be construed as a potential conflict of interest.

## Publisher’s note

All claims expressed in this article are solely those of the authors and do not necessarily represent those of their affiliated organizations, or those of the publisher, the editors and the reviewers. Any product that may be evaluated in this article, or claim that may be made by its manufacturer, is not guaranteed or endorsed by the publisher.
