# Supplementary material for: A multimodal meta-analysis of gray matter alterations in trigeminal neuralgia
Source: Front Neurol. 2023 Aug 3;14:1179896. doi: 10.3389/fneur.2023.1179896 (PMC10436096; doi:10.3389/fneur.2023.1179896)
Supplement: Supplementary file 1 [file Data_Sheet_1.pdf]

Supplementary materials

Methods

Multimodal Analysis

Table and Figure legends

Table S1. Quality assessment checklist (when criteria were partially met, 0.5 points assigned).

Table S2. Quality assessment scores of included studies.

Table S3. Gray matter alterations in patients with primary trigeminal neuralgia compared with healthy controls: robustness analyses.

Table S4. Brain functional alterations in patients with primary trigeminal neuralgia compared with healthy controls: robustness analyses.

Figure. S1. Funnel plot of Cluster 1 of increased gray matter volume in primary trigeminal neuralgia.

Figure. S2. Funnel plot of Cluster 1 of decreased gray matter volume in primary trigeminal neuralgia.

Figure. S3. Funnel plot of Cluster 2 of decreased gray matter volume in primary trigeminal neuralgia.

Figure. S4. Funnel plot of Cluster 3 of decreased gray matter volume in primary trigeminal neuralgia.

Figure. S5. Funnel plot of Cluster 4 of decreased gray matter volume in primary trigeminal neuralgia.

Figure. S6. Funnel plot of Cluster 1 of hyperactivation of brain response in primary trigeminal neuralgia.

Figure. S7. Funnel plot of Cluster 2 of hyperactivation of brain response in primary trigeminal neuralgia.

Figure. S8. Funnel plot of Cluster 3 of hyperactivation of brain response in primary trigeminal neuralgia.

Figure. S9. Funnel plot of Cluster 1 of hypoactivation of brain response in primary trigeminal neuralgia.

Figure. S10. Funnel plot of Cluster 2 of hypoactivation of brain response in primary trigeminal neuralgia.

Figure. S11. Funnel plot of Cluster 3 of hypoactivation of brain response in primary trigeminal neuralgia.

Methods

Multimodal Analysis

Areas of overlapping functional and structural abnormalities between patients with TN and healthy controls were assessed by conjunction analysis using the multimodal meta-analysis in AES-SDM. This multimodal meta-analysis approach aims to ensure that the false-positive rate is not increased compared with that in studies of any single modality[1]. We computed the intersection between meta-analysis results by multiplying the meta-analytic p-value maps. We obtained a probability map of gray matter (P_{GM}) and functional (P_F) alterations to identify regions with alterations in each modality using separate meta-analysis. The multimodal analysis combined the two probabilities maps, incorporating the P values to identify a union of alterations in both modalities (U). The estimation of U is straightforward as $U = P_{GM} + P_F - P_{GM} \times P_F$.. However, the U statistic in its raw form is overly conservative, and to reduce the imbalance between the false-positive and negative rates, U was adjusted according to $P = U+(1-U) \times \ln(1 - U)$ [1]. A more stringent probability threshold was employed for this multimodal analysis (p<0.0025) than that used in unimodal meta-analyses. It should be noted that this analysis did not aim to detect correlations between structural and functional abnormalities, but to localize brain regions in which TN is associated with both structural and functional alterations.

Table S1. Quality assessment checklist (when criteria were partially met, 0.5 points assigned).

**Category 1: Participants**<br>Score (0/0.5/1)
1. Patients were evaluated prospectively, specific diagnostic criteria were applied, and demographic data were reported.<br>2. Comparison participants were evaluated prospectively psychiatric and medical illnesses were excluded.<br>3. Important variables (e.g., age, sex, illness duration, onset, medication status, handedness) were checked either by stratification or statistically.<br>4. Sample size per group > 10.
**Category 2: Methods for image acquisition and analysis**
5. Whole brain analysis was automated with no *a priori* regional selection.<br>6. Coordinates reported in a standard space.<br>7. The imaging technique used was clearly described so that it could be reproduced.<br>8. Measurements were clearly described so that they could be reproduced.
**Category 3: Results and conclusions**
9. Statistical parameters for significant and important non-significant differences were provided.<br>10. Conclusions were consistent with the results obtained and the limitations were discussed.
TOTAL /10

Table S2. Quality assessment scores of included studies.

Studies	1	2	3	4	5	6	7	8	9	10	Total
VBM studies											
Gustin et al., 2011	1	0	1	1	1	1	1	1	1	0.5	8.5
Obermann et al., 2013	1	0	1	1	1	1	1	1	1	1	9
Li et al., 2017	1	0	1	1	1	1	1	1	1	1	9
Tsai et al., 2018	1	1	1	1	1	1	1	1	1	1	10
Zhang et al., 2018	1	1	1	1	1	1	1	1	1	1	10
Wang et.al., 2019	1	0	1	1	1	1	1	1	1	1	9
Wu et.al., 2020	1	0	1	1	1	1	1	1	1	1	9
Albano et al., 2022	1	1	1	1	1	1	1	1	1	1	10
Liu et al., 2022	1	1	1	1	1	1	1	1	1	1	10
Shen et.al., 2022	1	0	1	1	1	1	1	1	1	1	9
Functional studies											
Wang et al., 2015	1	1	1	1	1	1	1	1	1	1	10
Wang et al., 2017	1	0	1	1	1	1	1	1	1	0.5	8.5
Wang et al., 2017	1	1	1	1	1	1	1	1	1	1	10
Tsai et al., 2018	1	1	1	1	1	1	1	1	1	1	10
Yuan et al., 2018	1	1	1	1	1	1	1	1	1	1	10
Zhang et al., 2018	1	1	1	1	1	1	1	1	1	1	10
Chen et al., 2019	1	1	1	1	1	1	1	1	1	0.5	9.5
Xiang et al., 2019	1	1	1	1	1	1	1	1	1	0.5	9.5
Yan et al., 2019	1	1	1	1	1	1	1	1	1	1	10
Zhang et al., 2019	1	1	1	1	1	1	1	1	1	1	10
Zhu et al., 2020	1	1	1	1	1	1	1	1	1	1	10
Liu et al., 2022	1	1	1	1	1	1	1	1	1	1	10
Xu et al., 2022	1	1	1	1	1	1	1	1	1	1	10

Table S3. Structural alterations of gray matter in patients with primary trigeminal neuralgia compared with healthy controls: robustness analyses.

	GMV increase		GMV decrease							
	Right inferior temporal gyrus,	Right fusiform gyrus	Left insula	Left superior temporal gyrus	Left putamen	Left postcentral gyrus	Left inferior frontal gyrus	Bilateral thalamus	Right amygdala	Left striatum
Jackknife sensitivity analysis										
Gustin et al., 2011	Yes	Yes	Yes	Yes	Yes	Yes	Yes	Yes	No	Yes
Obermann et al., 2013	Yes	Yes	Yes	Yes	Yes	Yes	Yes	Yes	Yes	Yes
Li et al., 2017	Yes	Yes	Yes	Yes	Yes	Yes	Yes	Yes	No	Yes
Tsai et al., 2018	Yes	Yes	Yes	Yes	Yes	Yes	Yes	Yes	Yes	Yes
Zhang et al., 2018	Yes	Yes	Yes	Yes	Yes	Yes	Yes	Yes	No	Yes
Wang et.al., 2019	No	No	Yes	Yes	Yes	Yes	Yes	Yes	Yes	Yes
Wu et.al., 2020	Yes	Yes	Yes	Yes	Yes	Yes	Yes	Yes	Yes	Yes
Albano et al., 2022	Yes	Yes	Yes	Yes	Yes	Yes	Yes	Yes	Yes	Yes
Liu et al., 2022	Yes	Yes	Yes	Yes	Yes	Yes	Yes	Yes	Yes	Yes
Shen et.al., 2022	Yes	Yes	Yes	Yes	Yes	Yes	Yes	Yes	Yes	Yes
	9 out of 10	9 out of 10	10 out of 10	10 out of 10	10 out of 10	10 out of 10	10 out of 10	10 out of 10	7 of 10	10 out of 10
Subgroup analyses										
Studies using 3T scanners (n=11, 78%)	Yes	Yes	Yes	Yes	Yes	Yes	Yes	Yes	Yes	Yes
Studies using SPM software (n=13, 93%)	Yes	Yes	Yes	Yes	Yes	Yes	Yes	Yes	Yes	Yes
Studies applying 8 mm FWHM (n=12, 86%)	Yes	Yes	Yes	Yes	Yes	Yes	Yes	Yes	No	Yes

Abbreviations: SPM: statistical parametric mapping; FWHM: full width at half maximum.

Table S4. Functional alterations of gray matter in patients with primary trigeminal neuralgia compared with healthy controls: robustness analyses.

	Hyperactivity			Hypoactivity		
	Left cerebellum, hemispheric lobule VIII	Right thalamus	Left thalamus	Left middle temporal gyrus	Bilateral precuneus	Left superior temporal gyrus
Jackknife sensitivity analysis						
Wang et al., 2015	Yes	No	Yes	Yes	Yes	Yes
Wang et al., 2017	Yes	Yes	Yes	Yes	Yes	Yes
Wang et al., 2017	Yes	No	Yes	Yes	Yes	Yes
Tsai et al., 2018	Yes	Yes	Yes	Yes	Yes	Yes
Yuan et al., 2018	Yes	Yes	Yes	Yes	Yes	No
Zhang et al., 2018	Yes	Yes	Yes	Yes	Yes	Yes
Chen et al., 2019	No	Yes	Yes	Yes	Yes	Yes
Xiang et al., 2019	No	Yes	Yes	Yes	Yes	Yes
Yan et al., 2019	Yes	No	Yes	No	Yes	Yes
Zhang et al., 2019	Yes	Yes	No	Yes	Yes	Yes
Zhu et al., 2020	No	Yes	Yes	Yes	Yes	Yes
Liu et al., 2022	No	Yes	No	Yes	Yes	Yes
Xu et al., 2022	Yes	Yes	Yes	Yes	Yes	No
	10 out of 14	11 out of 14	12 out of 14	13 out of 14	14 out of 14	12 out of 14
Subgroup analyses						
Studies using 3.0T scanners (n=7, 78%)	No	No	Yes	Yes	Yes	Yes
Studies using SPM software (n=6, 67%)	Yes	Yes	No	Yes	Yes	No

Abbreviations: SPM: statistical parametric mapping.

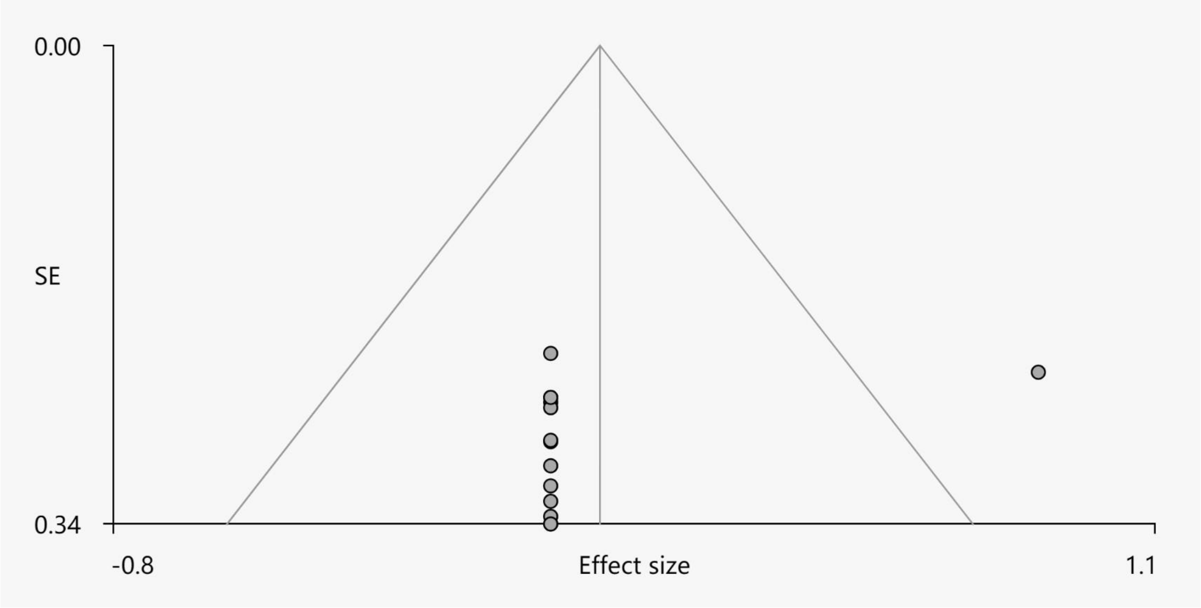

Figure. S1. Funnel plot of Cluster 1 of increased gray matter volume in primary trigeminal neuralgia.

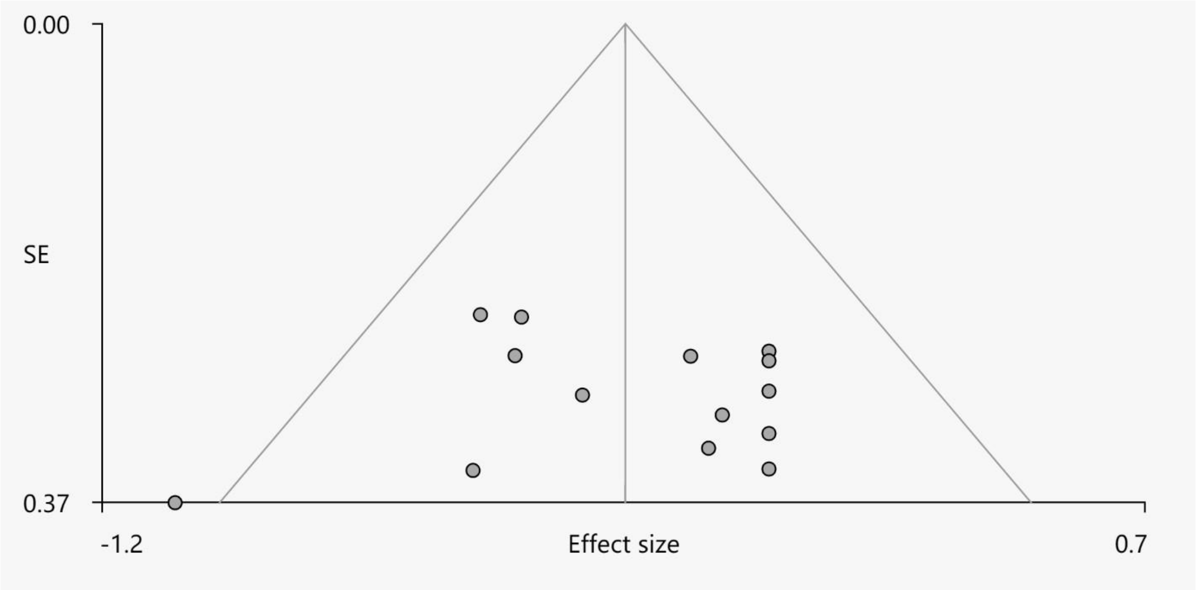

Figure. S2. Funnel plot of Cluster 1 of decreased gray matter volume in primary trigeminal neuralgia.

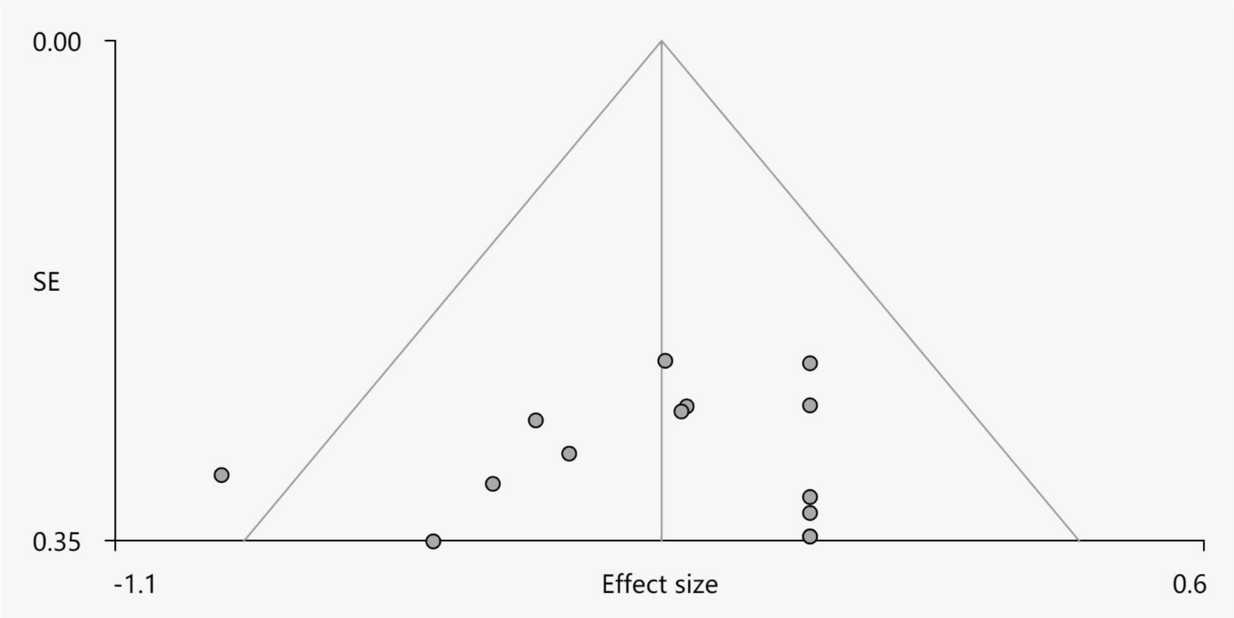

Figure. S3. Funnel plot of Cluster 2 of decreased gray matter volume in primary trigeminal neuralgia.

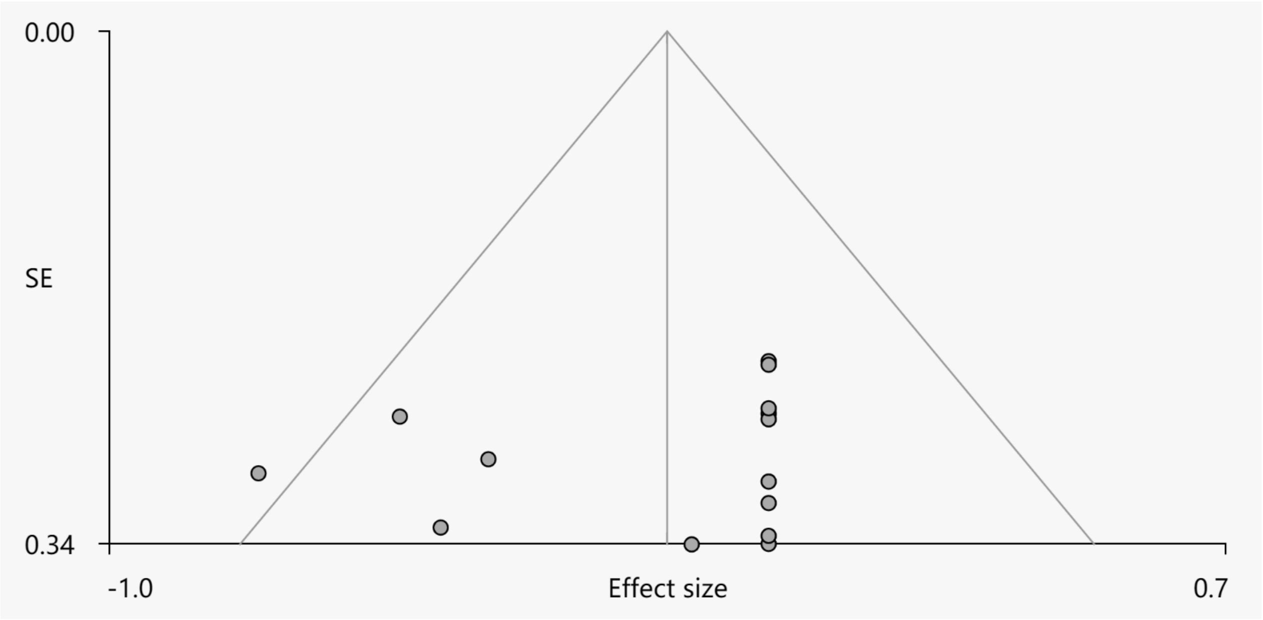

Figure. S4. Funnel plot of Cluster 3 of decreased gray matter volume primary trigeminal neuralgia.

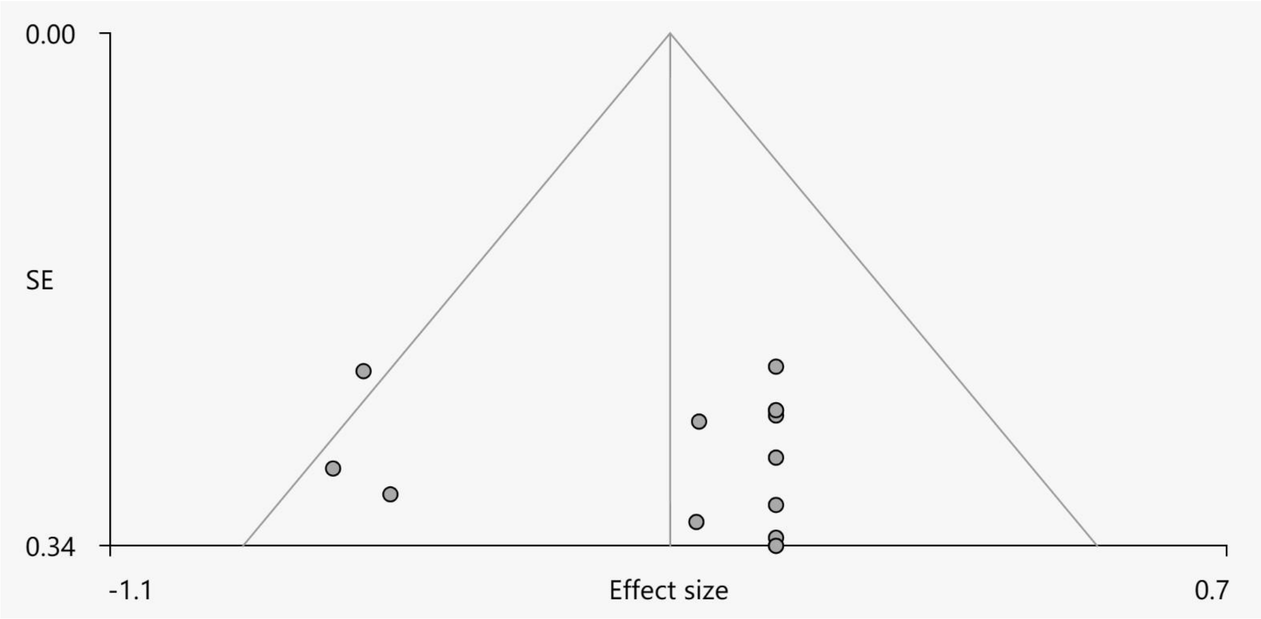

Figure. S5. Funnel plot of Cluster 4 of decreased gray matter volume in primary trigeminal neuralgia.

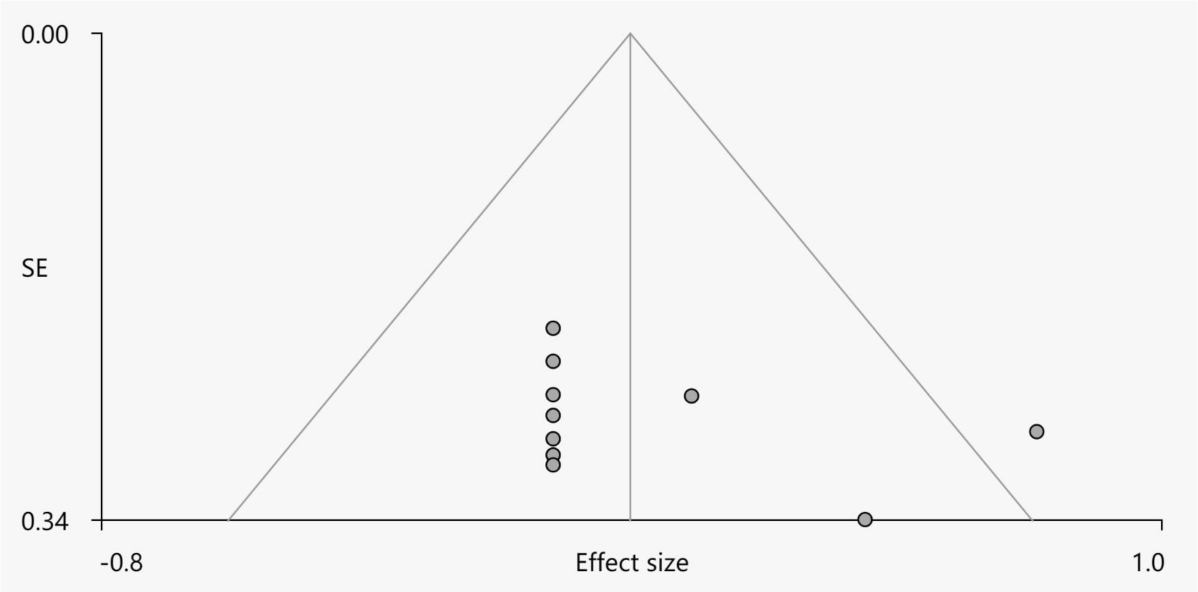

Figure. S6. Funnel plot of Cluster 1 of hyperactivation of brain response in primary trigeminal neuralgia.

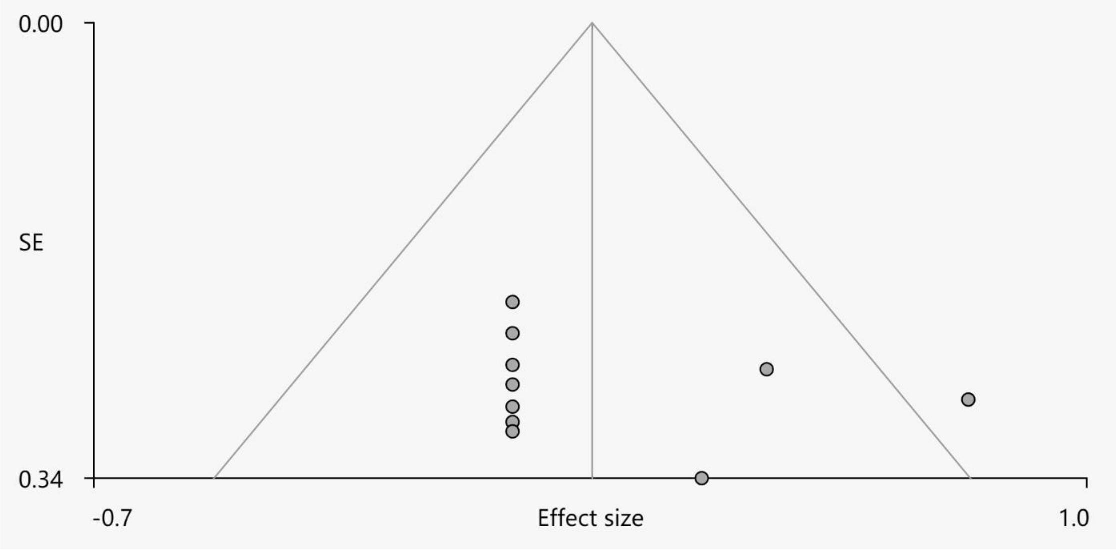

Figure. S7. Funnel plot of Cluster 2 of hyperactivation of brain response in primary trigeminal neuralgia.

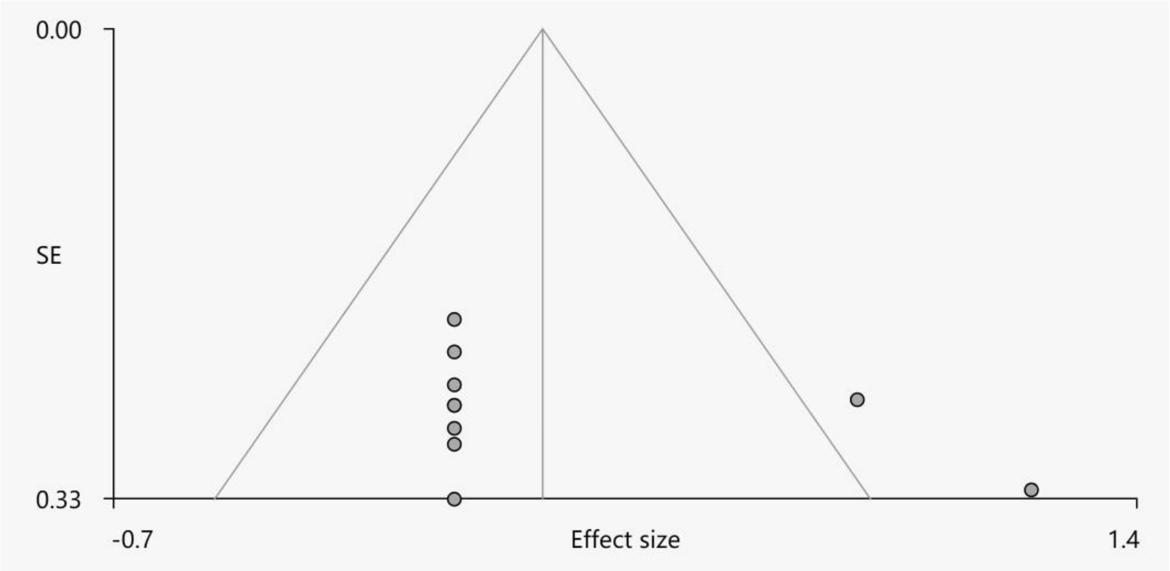

Figure. S8. Funnel plot of Cluster 3 of hyperactivation of brain response in primary trigeminal neuralgia.

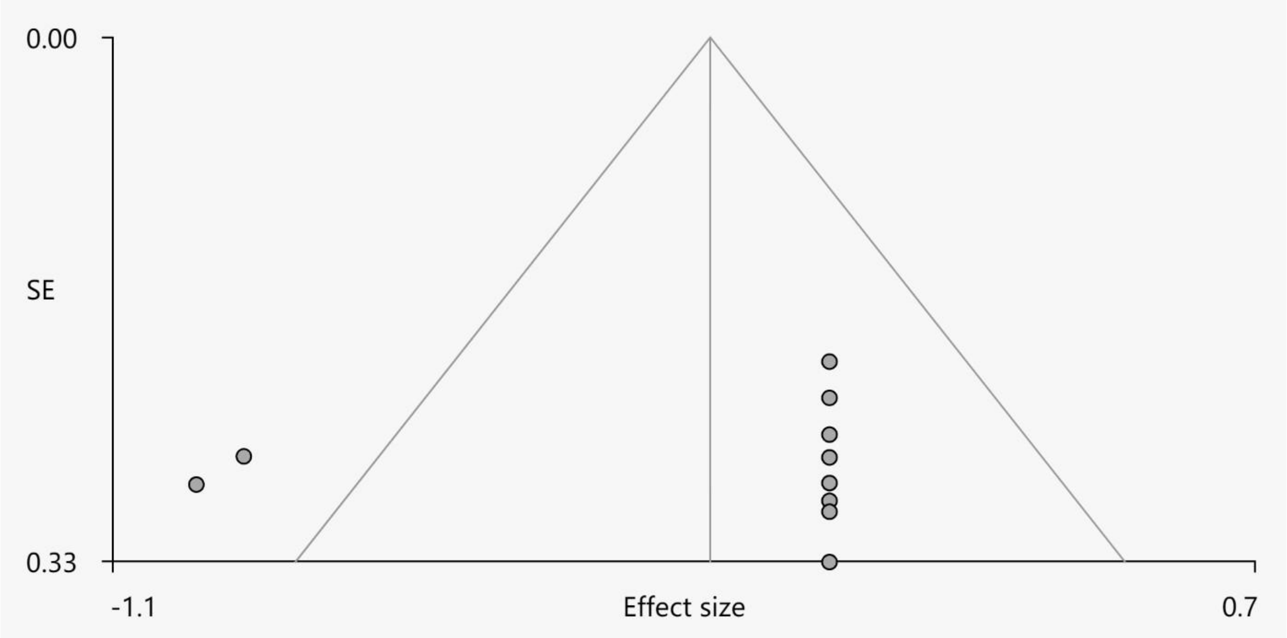

Figure. S9. Funnel plot of Cluster 1 of hypoactivation of brain response in primary trigeminal neuralgia.

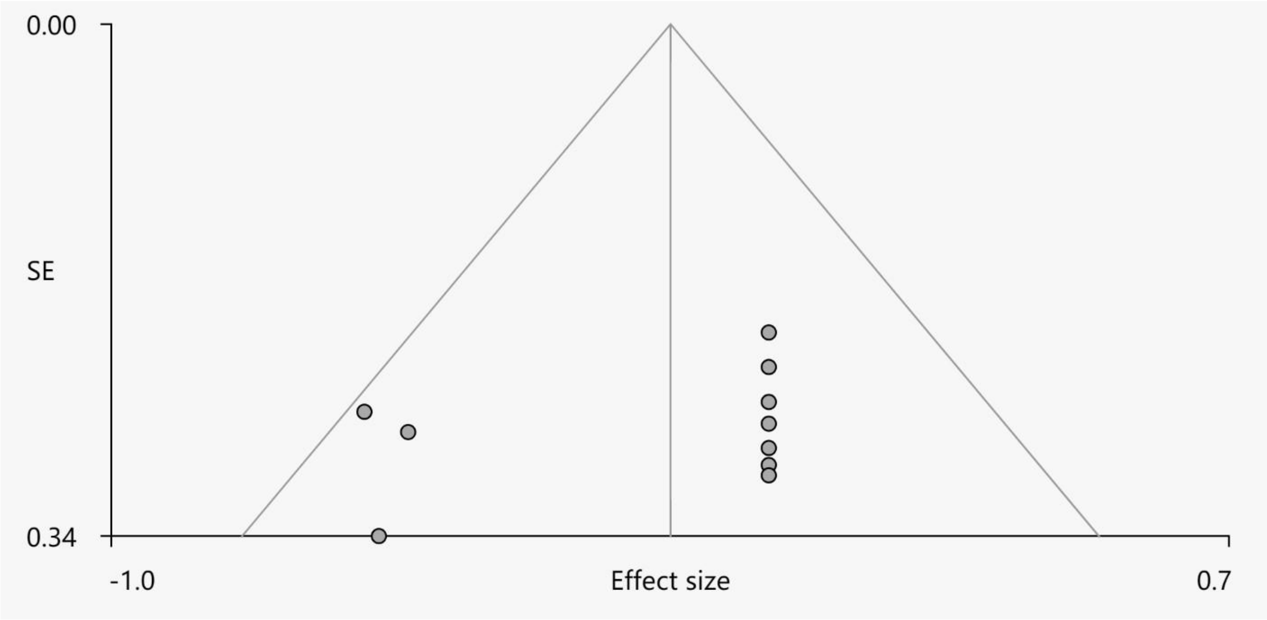

Figure. S10. Funnel plot of Cluster 2 of hypoactivation of brain response in primary trigeminal neuralgia.

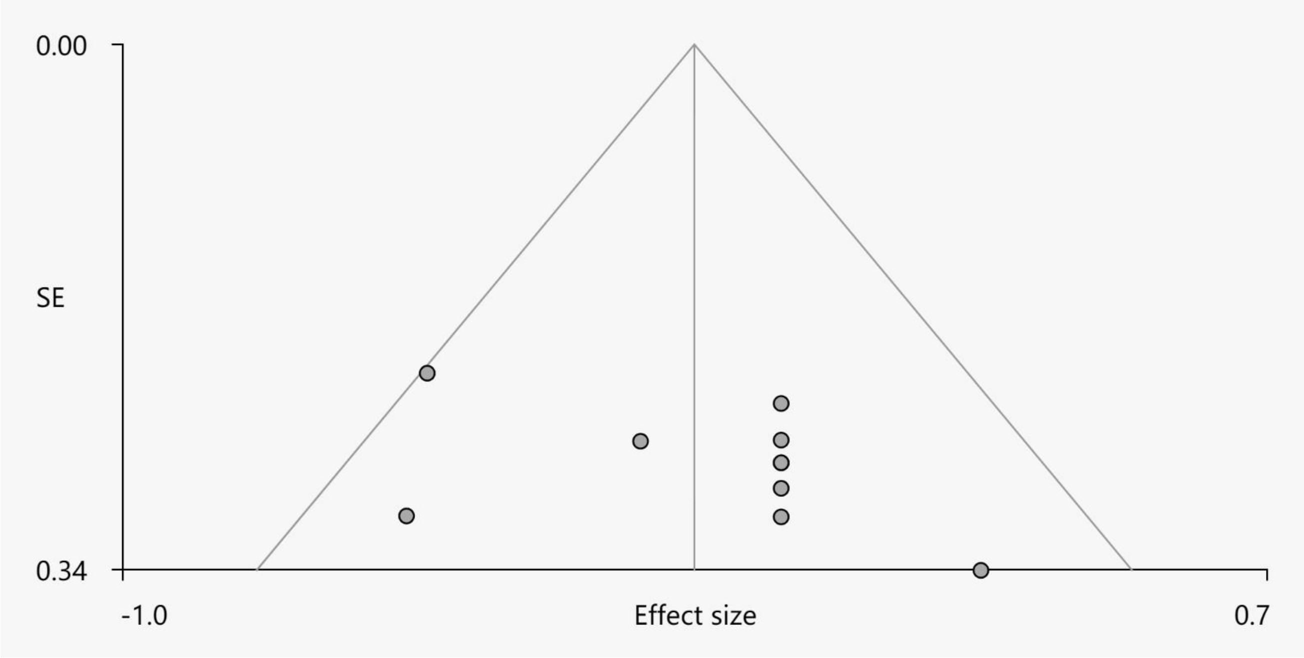

Figure. S11. Funnel plot of Cluster 3 of hypoactivation of brain response in primary trigeminal neuralgia.

References:

1. Radua J, Romeo M, Mataix-Cols D, Fusar-Poli P. A General Approach for Combining Voxel-Based Meta-Analyses Conducted in Different Neuroimaging Modalities. *Current medicinal chemistry* (2013) 20(3):462-6. Epub 2012/11/20.
